# Regular cannabis smoking and carotid artery calcification in the Multi-Ethnic Study of Atherosclerosis (MESA)

**DOI:** 10.1177/1358863X241287690

**Published:** 2024-10-23

**Authors:** Jamie Corroon, Ryan Bradley, Igor Grant, Michael R Daniels, Julie Denenberg, Michael P Bancks, Matthew A Allison

**Affiliations:** 1 Department of Family Medicine, University of California San Diego, La Jolla, CA, USA; 2 Herbert Wertheim School of Public Health and Human Longevity Science, University of California San Diego, La Jolla, CA, USA; 3 Department of Psychiatry, Center for Medicinal Cannabis Research, University of California San Diego, La Jolla, CA, USA; 4 Department of Epidemiology and Prevention, Wake Forest University School of Medicine, Winston-Salem, NC, USA

**Keywords:** atherosclerosis, cannabis, cardiovascular disease, carotid artery disease, marijuana

## Abstract

**Background:**

Studies on cannabis use and adverse cardiovascular outcomes have reported conflicting results. Research on its relationship to calcified arterial plaque remains limited.

**Methods:**

Cross-sectional data from 2152 participants at Exam 6 (2016–2018) in the Multi-Ethnic Study of Atherosclerosis (MESA) were analyzed, including self-reported cannabis smoking patterns and carotid artery calcification (CAC) as measured via computed tomography. Multivariable relative and absolute risk regression models were used to estimate adjusted prevalence ratios (PRs) and prevalence differences, respectively, for the presence of calcified plaque. Multivariable linear regression was then used to compare group differences in the extent of CAC in those with calcified plaque.

**Results:**

A minority of participants (*n* = 159, 7.4%) reported a history of regular cannabis smoking. Among all participants, 36.1% (*n* = 777) had detectable CAC. In models adjusted for demographics, behavioral, and clinical cardiovascular disease factors, a history of regular cannabis smoking was not associated with the prevalence of CAC in either common carotid artery (PR: 1.14, 95% CI: 0.88 to 1.49). In the subset of participants with calcified plaque, and in separate fully adjusted multivariable linear regression models, a history of regular cannabis smoking was not associated with increased calcium volume (difference = 7.7%, 95% CI: –21.8 to 48.5), calcium density (difference = 0.4%, 95% CI: –6.6 to 7.9), or Agatston score (difference = 32.1%, 95% CI: –31.8 to 155.8) in either carotid artery. Models exploring potential effect modification by age, race/ethnicity, and tobacco smoking status showed no significant association, except for higher CAC prevalence in men with a history of regular cannabis smoking.

**Conclusions:**

In a racially and ethnically diverse cohort of older adults with a moderately high prevalence of CAC, no associations were found between a history of regular cannabis smoking, duration, or recency of cannabis smoking, and the prevalence of carotid calcified plaque. These findings were consistent across age, race/ethnicity, and cigarette smoking, except for an increased prevalence in men with a history of regular cannabis smoking. Similarly, in a subgroup with CAC, no association was found between a history of regular cannabis smoking and extent of calcification as measured by volume, density, and Agatston score.

## Background

In recent years, the increasing use of cannabis (*Cannabis sativa* L., marijuana), driven by its broader legalization and acceptance for medical purposes, has resulted in a growing interest in its health implications.^1,2^ Unlike tobacco, whose negative cardiovascular effects are well-recognized,^3^ the cardiovascular effects of cannabis, particularly its association with atherosclerosis, remain a subject of evolving research.^4^

Carotid artery calcification (CAC) is an established independent risk factor for heart disease, stroke, and myocardial infarction (MI).^5–7^ Although research that measures the prevalence of calcified plaque shows a lower prevalence in the carotid arteries than in other vascular beds (i.e., carotid: 32.2% vs coronary: 55.8%), calcification in the carotid arteries may indicate a more extensive atherosclerotic burden, and is associated with a higher risk of both total and noncardiovascular disease mortality compared to calcification in the coronary arteries.^8^

Despite cross-sectional evidence suggesting an association between chronic cannabis use and higher prevalence of atherosclerosis and other cardiovascular diseases (CVDs),^9–11^ including stroke,^12,13^ the evidence is conflicting, and there remains a gap in determining whether cannabis use is associated with calcified plaque in the carotid arteries specifically. Notably, two published longitudinal studies failed to detect associations between lifetime cannabis use and carotid intima–media thickness^14^ or coronary or abdominal artery calcium in never tobacco smokers.^15^ Because of the strong predictive value of calcified plaque on CV events, this discrepancy underscores the need for further research to clarify the relationship between regular cannabis use and cardiovascular health, and may help elucidate mechanisms through which cannabis affects risk.

As such, the research presented here aimed to investigate associations between regular cannabis smoking and calcified plaque in the carotid arteries in a large, multiethnic cohort of US adults without history of CVD at baseline.

## Methods

### Study population

The Multi-Ethnic Study of Atherosclerosis (MESA) is a prospective cohort study of men and women aged 45–84 years who were free of clinical CVD at the time of study enrollment (*n* = 6814). Participants were recruited from 2000 to 2002 at six US field centers from four race/ethnic groups: non-Hispanic White (38%), non-Hispanic Black/African American (28%), Hispanic American (22%), and Chinese Americans (12%). All participants provided written informed consent. The original MESA study was approved by the institutional review board of each field center. Detailed methodology for MESA has been published previously.^16^

### Analytic sample

The analytic sample (*n* = 2152) was limited to all MESA participants with complete data for two questions used to define a history of regular cannabis use and lung CT scans scored for the presence of calcium in carotid arteries at Exam 6 (2016–2018).

### Independent variables

#### Cannabis use

At Exam 6, participants responded to a series of questions about cannabis use (Supplementary Figure 1). Participants classified as having a history of regular cannabis smoking were those who responded ‘Yes’ to the questions: (1) ‘Have you smoked more than 100 marijuana or hashish joints/pipes in your life?’ and (2) ‘Have you ever smoked marijuana or hashish regularly (at least once per month)?’ Those classified as having no history of regular smoking were those who reported ‘Yes’ or ‘No’ to the first question and ‘No’ to the second.

Participants with a history of regular smoking were subsequently asked: ‘For how many years did you smoke marijuana or hashish regularly?’ and ‘How long has it been since you last smoked marijuana or hashish?’. Years of regular smoking were analyzed as both continuous and categorical variables. Recency of smoking was defined as any reported cannabis smoking within the preceding 31 days (Supplementary Figure 1).

To assess a multifaceted exposure including duration, frequency, and quantity of cannabis smoking, a composite measure was created (i.e., joint/pipe years). Joint/pipe years was calculated using the midpoint for each categorical response to ‘During the time that you smoked marijuana or hashish regularly, how often would you usually smoke it?’ (Supplementary Methods). Joint/pipe years was calculated as follows: (midpoint value of each categorical response to ‘During the time that you smoked marijuana or hashish regularly, how often would you usually smoke it?’ × 12 months/year × ‘On the days that you smoked marijuana or hashish, how many joints or pipes would you usually smoke?’ × years of regular smoking) / days per year. For example: (6 times per month × 12 months/year × 3 joints/pipes per day × 10 years of regular smoking) / 365.25 = 5.9 joint/pipe years.

#### Covariates

Site, sex, race/ethnicity, household income, and educational attainment were assessed at the Baseline Exam, whereas all other variables were assessed at Exam 6, including age, physical activity, cigarette smoking, current alcohol use, and medication use. Height, weight, and biomarkers (total cholesterol [mg/dL], high-density lipoprotein [HDL] cholesterol [mg/dL], fasting blood glucose [mg/dL], and HbA1c [%]) were objectively measured at Exam 6 in the central laboratory (University of Vermont, Burlington, VT, USA) using standardized protocols and calibrated measurements. Also at this visit, resting seated systolic (SBP) and diastolic (DBP) blood pressure were measured in triplicate at 1-minute intervals. Readings were averaged to obtain mean SBP and DBP.

Dyslipidemia was defined as a total cholesterol to HDL ratio > 5.0 or use of lipid-lowering medication. Prediabetes was defined as fasting blood glucose 100–125 mg/dL or Hb A1c 5.7–6.4%. Diabetes was defined as fasting blood glucose ⩾ 126 mg/dL, HbA1c ⩾ 6.5%, or the use of antidiabetic medication.

#### Dependent variables

The methodology for acquisition and interpretation of calcified atherosclerosis in the carotid arteries using lung computed tomography (CT) scans, as well as the reproducibility of the readings, have been reported previously.^17^ Lung CT scans were obtained as part of an ancillary study within MESA for 2381 individuals at visit 6 and 2170 CT scans (91%) were scored for the presence of calcium.

Carotid artery calcium was assessed using either an electron-beam CT scanner (Chicago, Los Angeles, and New York) or a multidetector CT system (Baltimore, St Paul, and Winston-Salem). All scanners were cardiac-gated. Slice thickness was 3 mm using electron-beam CT scanners and 2.5 mm using multidetector CT scanners. The scans were evaluated in a blinded manner by technologists specifically trained for this task, operating outside of the CT Reading Center at the University of California, San Diego. To quantify the calcium scores, Philips (Cardiac) Calcium Scoring (Heartbeat CS) software was used. This software calculates the total calcium volume in an artery or arterial segment by multiplying the lesion area in mm^3^ (each lesion on each slice above the 130 Hounsfield units (HU) threshold) by the slice thickness, set at 0.5 mm. Calcium was quantified using the method described by Agatston et al.^18^

The right and left common carotid arteries were identified from their origins from the brachiocephalic trunk and thoracic aorta, respectively. Volume, density, and Agatston values from the right brachiocephalic trunk and right common carotid artery were combined to give the presence/absence and extent of calcium in the right common carotid.

#### Statistical methods

Descriptive statistics were used to characterize the sample. Means and SDs were reported for continuous variables, whereas counts and percentages were used for nominal variables. Continuous variables were assessed for plausibility (Supplementary Methods).

The primary analysis evaluated associations between a history of regular cannabis smoking and the prevalence and extent of calcified plaque in either carotid artery (primary outcome: presence/absence and extent of CAC). Secondary analyses included stratified analyses for the primary analysis by age, sex, race/ethnicity, and tobacco smoking status, as well as assessment of associations of the duration of monthly cannabis smoking and recency of cannabis smoking.

Exploratory analyses included a composite variable (i.e., joint/pipe years) to evaluate a multifaceted exposure to cannabis that included duration, frequency, and quantity.

Multivariable relative and absolute risk regression models with robust standard errors were used to estimate adjusted prevalence ratios (PRs) and prevalence differences (PDs), respectively, with 95% CIs for the prevalence of calcified plaque, controlling for potential confounders. Multivariable linear regression was then used to assess the extent of carotid artery calcium in those with calcified plaque. Extent was measured as volume (mm^3^), density (HU), and Agatston score. These variables were natural log transformed due to skew. Resulting beta coefficients were exponentiated for interpretation. Multivariable linear regression was also used in secondary and exploratory analyses to evaluate the duration of regular smoking in years and the number of joints or pipes smoked per day as continuous variables.

Variables considered as potential confounders in the multivariable analyses were: sociodemographic variables including site, age, sex, race/ethnicity, education level, and household income (model 1); behavioral risk factors such as cigarette smoking, alcohol use, and physical activity (model 2); and body mass index (BMI) and CVD risk factors, including SBP, DBP, total cholesterol to HDL ratio, fasting blood glucose (FBG), and antilipidemic, antidiabetic, antihypertensive and total medications (model 3). For linear regression models assessing extent of calcified plaque as the dependent variable, a fourth model adjusted *volume* for density and *density* for volume.

Assessment of collinearity between independent variables was performed in the final multivariable adjusted model using the variance inflation factor and tolerance. Statistical significance was set at *p* < 0.05.

#### Stratified analyses

Stratified analyses were used to assess differences between a history of regular cannabis smoking and the prevalence of CAC in age-defined subgroups (i.e., 59–69, 70–80, > 80 years), race/ethnicity-defined subgroups (i.e., non-Hispanic White, non-Hispanic Black, Hispanic, Chinese), subgroups by biologic sex (i.e., men, women), and subgroups based on cigarette smoking status (i.e., current, former, never). Multiplicative first-order interactions were constructed for each in the final model. Interaction terms were considered significant in the final multivariable-adjusted model if *p* < 0.10.

## Results

The study sample included 2152 participants, a small minority of whom (*n* = 159, 7.4%) reported a history of regular cannabis smoking (Table 1). Those with such a history were generally younger, more likely to be men, non-Hispanic Black, engage in more risky behaviors (higher alcohol intake, higher mean pack-year history of cigarette smoking, and higher prevalence of current cigarette smoking), but also report more physical activity than those without a history of regular cannabis smoking. Notably, they also had a lower prevalence of hypertension, dyslipidemia, diabetes, and prediabetes, and a similar BMI compared to participants without a history of regular cannabis smoking. Compared to the overall cohort, the analytic sample had a statistically significantly higher prevalence of never-tobacco smokers and a greater median met-minutes per week of physical activity.

**Table 1.** Characteristics of participants by history of regular cannabis smoking (*n* = 2152).

	Overall sample	No history of regular cannabis smoking	History of regular cannabis smoking
Characteristic	(*n* = 2152, 100.0%)	^(n = 1993, 92.6%)^	(*n* = 159, 7.4%)
**Sociodemographics**			
Age, years, mean (SD)	73.5 (8.5)	74.0 (8.5)	67.5 (5.7)
Men, *n* (%)	1003 (46.6)	896 (45.0)	107 (67.3)
**Race/ethnicity, *n* (%)**			
Non-Hispanic White	826 (38.4)	762 (38.2)	64 (40.3)
Non-Hispanic Black	557 (25.9)	486 (24.4)	71 (44.7)
Hispanic	461 (21.4)	438 (22.0)	23 (14.5)
Chinese	308 (14.3)	307 (15.4)	1 (0.6)
**Education, *n* (%)**			
< Bachelor’s degree	1247 (58.1)	1167 (58.7)	80 (50.3)
Bachelor’s degree	409 (19.1)	371 (18.7)	38 (23.9)
Graduate or professional school	490 (22.8)	449 (22.6)	41 (25.8)
**Gross family income, *n* (%)**			
⩽ $49,999	1049 (50.5)	994 (51.8)	55 (34.8)
$50,000 to $99,999	587 (28.3)	527 (27.5)	60 (38.0)
⩾ $100,000	441 (21.2)	398 (20.7)	43 (27.2)
**Lifestyle, behavioral**			
Alcohol intake, drinks/week, mean (SD)	0.0 (0.0, 2.0)	0.0 (0.0, 2.0)	0.0 (0.0, 7.0)
Cigarette smoking status, *n* (%)			
Never	1065 (49.5)	1029 (51.6)	36 (22.6)
Former	985 (45.8)	881 (44.2)	104 (65.4)
Current	102 (4.7)	83 (4.2)	19 (12.0)
Cigarette smoking, pack years, mean (SD)	8.3 (17.0)	7.8 (16.4)	15.6 (22.4)
Cigarette smoking, pack years, median (IQR)	0.0 (0.0, 18.0)	0.0 (0.0, 7.5)	6.2 (0.2, 23.5)
Physical activity, met-min/week, median (IQR)	2820.0 (1215.0, 5910.0)	3240.0 (1395.0, 6660.0)	5445.0 (2595.0, 10,102.5)
BMI, mean (SD)	28.8 (5.8)	28.8 (5.8)	28.8 (5.9)
**Cardiometabolic medical history**			
Systolic BP, mmHg, mean (SD)	128 (21)	128 (21)	125 (19)
Diastolic BP, mmHg, mean (SD)	69 (10)	69 (10)	72 (11)
Hypertension, *n* (%)	1596 (74.2)	1484 (74.5)	112 (70.4)
Dyslipidemia, *n* (%)	1136 (52.8)	1060 (53.2)	76 (47.8)
Diabetes or prediabetes, *n* (%)	1230 (57.2)	1148 (57.6)	82 (51.6)
Carotid artery calcium, *n* (%)	777 (36.1)	729 (36.6)	48 (30.2)
Agatston score^a^, mean (IQR)	40.4 (127.5)	39.9 (127.5)	42.2 (115.5)
**Medications, *n* (%)**			
Antihypertensive	1311 (60.9)	1226 (61.5)	85 (53.5)
Antilipidemic	1020 (47.4)	949 (47.6)	71 (44.7)
Antidiabetic	415 (19.3)	397 (19.9)	18 (11.3)
Total medications, mean (SD)	7.1 (4.7)	7.1 (4.7)	7.0 (4.9)

a Among those with any carotid artery calcium; left and right carotid arteries combined.

BMI, body mass index; BP, blood pressure; met-min/week, metabolic equivalent of task minutes per week; mmHg, millimeters mercury.

### Characteristics of cannabis use

Among those who reported smoking 100 or more joints/pipes in their lifetime, a majority (80.3%) reported a history of regular use (smoking at least once per month) (Table 2). The mean duration of regular cannabis smoking was 16.6 years among this group, with non-Hispanic Black participants reporting a slightly longer mean duration (18.6 years). More than half of those with a history of regular use reported a frequency of use of once a week or more (56.6%). Daily use was reported by 22.6% of those with a history of regular use and nearly 40% of Hispanic participants with such a history. Nearly one-third of those with a history of regular use reported smoking more than one joint/pipe per day. Slightly less than 3% of the overall sample, and slightly less than 40% of participants with a history of regular smoking, reported smoking cannabis in the past month (recent use).

**Table 2.** Cannabis smoking characteristics of participants with a history of regular use by race/ethnicity (*n* = 159).

	Overall sample	Non-Hispanic White	Non-Hispanic Black	Hispanic	Chinese
Characteristic	(*n* = 159, 100.0%)	(*n* = 64, 40.3%)	(*n* = 71, 44.7%)	(*n* = 23, 14.5%)	(*n* = 1, 0.6%)
**History of regular use, yes, *n* (%)**					
Among all participants	159 (7.4)	64 (7.8)	71 (12.8)	23 (5.0)	1 (0.3)
Among those with ⩾ 100 joints/pipes lifetime	159 (80.3)	64 (80.0)	71 (80.7)	23 (82.1)	1 (50.0)
**Duration of regular use, mean (SD)**					
Years (*n* = 127)	16.6 (15.5)	15.3 (14.8)	18.6 (17.0)	15.3 (13.0)	5.0 (0.0)
**Frequency of regular use, n (%)**					
< Once a week	25 (15.7)	11 (17.2)	9 (12.7)	5 (21.7)	0 (0.0)
⩾ Once a week	90 (56.6)	38 (59.4)	43 (60.6)	8 (34.8)	1 (100.0)
Daily	36 (22.6)	12 (18.8)	15 (21.1)	9 (39.1)	0 (0.0)
Missing/refused/don’t know	8 (5.0)	3 (4.7)	4 (5.6)	1 (4.4)	0 (0.0)
**Quantity of daily use, *n* (%)**					
⩽ 1 joints/pipes	71 (44.7)	33 (51.6)	30 (42.3)	8 (34.8)	0 (0.0)
> 1 joints/pipes	52 (32.7)	14 (21.9)	28 (39.4)	10 (43.5)	0 (0.0)
Missing/refused/don’t know	36 (22.6)	17 (26.6)	13 (18.3)	5 (21.7)	1 (100.0)
**Duration, frequency, and quantity, median (IQR)**					
Joint/pipe years (midpoint, *n* = 151)	6.0 (6.0, 16.5)	6.0 (6.0, 16.5)	6.0 (16.5, 10.5)	11.3 (6.0, 27.5)	6.0 (6.0, 6.0)
**Recency of use, *n* (%)**					
Past month use (*n* = 151)					
Among all participants	60 (2.8)	40 (3.1)	37 (4.5)	4 (0.6)	0 (0.0)
Among those with history of regular use	60 (39.7)	40 (40.4)	37 (38.5)	4 (14.3)	0 (0.0)

### Prevalence of carotid artery calcium

Among all participants, 36.1% (*n* = 777) had detectable CAC (Table 1). When compared to participants without a history of regular cannabis smoking, those with a history had a lower prevalence of calcified plaque (volume > 0) in the left, right, and both common carotid arteries (Table 3). For scores greater than 0, the prevalence was generally lower at each category of Agatston score, except for a score ⩾ 300 for the left common carotid.

**Table 3.** Frequency and prevalence (unadjusted) for left, right, and both common carotid arteries by Agatston score category and history of regular cannabis smoking.

Agatston score	No history of regular cannabis smoking			History of regular cannabis smoking		
	Left	Right	Both	Left	Right	Both
**0**	1620 (81.3)	1457 (73.1)	1333 (66.9)	138 (86.8)	125 (78.6)	116 (73.0)
**1–99**	323 (16.2)	348 (17.5)	425 (21.3)	17 (10.7)	22 (13.8)	30 (18.9)
**100–299**	45 (2.3)	124 (6.2)	144 (7.2)	2 (1.3)	9 (5.7)	7 (4.4)
**⩾ 300**	5 (0.3)	64 (3.2)	91 (4.6)	2 (1.3)	3 (1.9)	6 (3.8)

Both is the sum of the, left and right carotid artery Agatston scores.

In unadjusted relative and absolute risk regression models, a history of regular cannabis smoking compared with no history was not significantly associated with relative or absolute prevalence of calcified plaque in either carotid artery (primary outcome) (PR: 0.83, 95% CI: 0.65 to 1.05; PD: –6.4%, 95% CI: –13.8 to 1.1) (Table 4). In fully adjusted models, the prevalence ratio (PR: 1.14, 95% CI: 0.88 to 1.49) and prevalence difference (PD: 4.7%, 95% CI: –2.7 to 12.0) were accentuated. Both findings remained nonstatistically significant.

**Table 4.** Adjusted prevalence ratios and absolute prevalence differences for calcified plaque in either carotid artery by cannabis smoking characteristics.

Cannabis characteristic		Unadjusted		Fully adjusted	
	(Prevalence of calcified plaque)	Prevalence ratio	Prevalence difference	Prevalence ratio	Prevalence difference
**History of regular smoking**		(*n* = 2152)		(*n* = 1997)	
No	729/1993 (36.6%)	**1.00 Ref.**	**0.00 Ref.**	**1.00 Ref.**	**0.00 Ref.**
Yes	48/159 (30.2%)	0.83 (0.65 to 1.05)	–6.4 (–13.8 to 1.1)	1.14 (0.88 to 1.49)	4.7 (–2.7 to 12.0)
**Duration of regular smoking, years**		(*n* = 2120)		(*n* = 1967)	
0	729/1993 (36.6%)	**1.00 Ref.**	**0.00 Ref.**	**1.00 Ref.**	**0.00 Ref.**
1 to 5	14/47 (29.8%)	0.81 (0.52 to 1.27)	–6.8 (–20.0 to 6.5)	1.35 (0.91 to 2.01)	7.9 (–4.0 to 19.8)
> 5 to 10	7/26 (26.9%)	0.74 (0.39 to 1.39)	–9.7 (–26.8 to 7.5)	1.42 (0.90 to 2.23)	4.6 (–10.0 to 19.3)
> 10	17/54 (31.5%)	0.86 (0.58 to 1.28)	–5.1 (–17.7 to 7.5)	1.00 (0.64 to 1.56)	2.1 (–10.4 to 14.7)
**Recency of smoking**		(*n* = 2144)		(*n* = 1989)	
No history of regular smoking	729/1993 (36.6%)	**1.00 Ref.**	**0.00 Ref.**	**1.00 Ref.**	**0.00 Ref.**
No past month smoking	30/91 (33.0%)	0.90 (0.67 to 1.21)	–3.6 (–13.5 to 6.3)	1.19 (0.86 to 1.65)	6.3 (–2.9 to 15.4)
Past month smoking	15/60 (25.0%)	0.68 (0.44 to 1.06)	–11.6 (–22.7 to –0.4)	1.04 (0.68 to 1.61)	1.2 (–10.3 to 12.7)

All comparisons with participants who reported no history of regular smoking.

Fully adjusted model: site, age, sex, race/ethnicity, HH income, alcohol and cigarette use, physical activity, BMI, SBP, DBP, total cholesterol to HDL cholesterol ratio, FBG, antihypertensive, antilipidemic, antidiabetic, and total medications.

The *p*-values for multiple comparisons have been adjusted using Tukey’s method.

Bold = *p* < 0.05.

BMI, body mass index; DBP, diastolic blood pressure; FBG, fasting blood glucose; HDL, high density lipoprotein; HH, household; SBP, systolic blood pressure.

The duration of regular cannabis smoking was not associated with CAC in unadjusted or fully adjusted models. Though an exposure–response relationship was absent in the unadjusted model, a lower prevalence difference was found with greater duration of cannabis smoking in the fully adjusted model.

Cannabis smoking in the past month (recency) by participants with a history of regular smoking was not significantly associated with CAC in unadjusted (PR: 0.68, 95% CI: 0.44 to 1.06; PD: –11.6%, 95% CI: –22.7 to –0.4) or fully adjusted models (PR: 1.04, 95% CI: 0.68 to 1.61; PD: 1.2%, 95% CI: –10.3 to 12.7).

### Extent of carotid artery calcium

In fully adjusted multivariable linear regression models in the subset of participants with calcified plaque (i.e., volume > 0), a history of regular smoking was not significantly associated with increased calcium volume (difference = 7.7%, 95% CI: –21.8 to 48.5), calcium density (difference = 0.4%, 95% CI: –6.6 to 7.9), or Agatston score (difference = 32.1%, 95% CI: –31.8 to 155.8) in either carotid artery (Table 5). Similarly, neither duration of regular cannabis smoking nor the number of joint/pipe years was significantly associated with any of the three outcome measures.

**Table 5.** Adjusted percent differences and 95% confidence intervals for extent of calcified plaque in carotid arteries by cannabis smoking characteristics.

Cannabis characteristic	Unadjusted			Fully adjusted		
	Volume (mm^3^) % difference (95% CI)	Density (HU) % difference (95% CI)	Agatston score % difference (95% CI)	Volume (mm^3^) % difference (95% CI)	Density (HU) % difference (95% CI)	Agatston score % difference (95% CI)
**History of regular smoking, y/n**						
Left common carotid	22.8 (–33.8 to 127.8)	3.0 (–13.1 to 22.1)	26.1 (–41.9 to 173.7)	13.7 (–23.1 to 68.2)	1.4 (–8.9 to 12.8)	81.4 (–20.4 to 313.5)
	(*n* = 447)	(*n* = 447)	(*n* = 447)	(*n* = 415)	(*n* = 415)	(*n* = 415)
Right common carotid	–13.5 (–50.7 to 51.7)	–0.7 (–13.0 to 13.3)	–19.7 (–59.4 to 58.6)	10.9 (–22.2 to 58.1)	–1.5 (–9.4 to 7.1)	12.2 (–47.0 to 137.4)
	(*n* = 625)	(*n* = 625)	(*n* = 625)	(*n* = 574)	(*n* = 574)	(*n* = 574)
Either common carotid	–11.0 (–46.9 to 49.0)	0.0 (–10.7 to 12.0)	–14.4 (–54.0 to 59.5)	7.7 (–21.8 to 48.5)	0.4 (–6.6 to 7.9)	32.1 (–31.8 to 155.8)
	(*n* = 777)	(*n* = 777)	(*n* = 777)	(*n* = 715)	(*n* = 715)	(*n* = 715)
**Duration of regular smoking, years**						
Either common carotid	–0.2 (–1.7 to 1.2)	0.2 (–0.1 to 0.5)	1.9 (–1.0 to 5.0)	0.0 (–1.4 to 1.5)	0.1 (–0.2 to 0.4)	1.5 (–1.6 to 4.6)
	(*n* = 731)	(*n* = 731)	(*n* = 731)	(*n* = 706)	(*n* = 706)	(*n* = 706)
**Joint/pipe years, mid**						
Either common carotid	1.1 (–0.4 to 2.7)	1.5 (0.0 to 3.1)	1.3 (–0.6 to 3.2)	0.7 (–0.2 to 1.6)	–0.1 (–0.3 to 0.1)	1.7 (–0.1 to 3.5)
	(*n* = 760)	(*n* = 760)	(*n* = 760)	(*n* = 700)	(*n* = 700)	(*n* = 700)

Percent change in volume, density, and Agatston score was calculated after natural log transformation and then back-transformed.

Fully adjusted model: site, age, sex, race/ethnicity, HH income, volume (mm^3^) for density, density (HU) for volume, alcohol and cigarette use, physical activity, BMI, SBP, DBP, total cholesterol to HDL cholesterol ratio, FBG, antihypertensive, antilipidemic, antidiabetic, and total medications.

Bold = *p* < 0.05.

BMI, body mass index; DBP, diastolic blood pressure; FBG, fasting blood glucose; HDL, high density lipoprotein; HH, household; HU, Hounsfield units; SBP, systolic blood pressure.

### Stratified analyses

When stratified by sex, the prevalence of calcified plaque in either carotid artery was significantly higher among men with a history of regular cannabis smoking (PR: 1.36, 95% CI: 1.06 to 1.75) compared to no history among men. Otherwise, models exploring the potential effect measure modification between a history of regular cannabis smoking and age, race/ethnicity, and cigarette smoking status did not support differential associations (Table 6).

**Table 6.** Adjusted prevalence ratios and 95% confidence intervals for calcified plaque in either carotid artery, stratified by selected characteristics (*n* = 2000).

Characteristic	History of regular cannabis smoking (Yes)	*p* for interaction
**Age, y**		0.56
59–69	1.13 (0.81 to 1.56)	
70–80	1.18 (0.76 to 1.81)	
> 80	0.86 (0.38 to 1.98)	
**Sex**		0.31
Male	**1.36 (1.06 to 1.75)**	
Female	0.83 (0.44 to 1.57)	
**Race/ethnicity**		0.64
Non-Hispanic White	1.16 (0.81 to 1.66)	
Non-Hispanic Black	0.93 (0.59 to 1.47)	
Hispanic	1.21 (0.74 to 1.97)	
Chinese	NA	
**Cigarette smoking status**		0.22
Never	1.02 (0.50 to 2.07)	
Former	0.90 (0.62 to 1.31)	
Current	1.27 (0.81 to 2.00)	

Reference = no history of regular cannabis smoking.

Statistically significant prevalence ratios in bold.

Interaction *p*-value calculated with one interaction term in the fully adjusted model; *p*-values for multiple comparisons have been adjusted using Tukey’s method.

NA, not applicable.

## Discussion

In a racially and ethnically diverse cohort of older adults with a moderately high prevalence of CAC, there were no statistically significant associations between a history of regular cannabis smoking (primary analysis), duration, or recency of cannabis smoking, and the prevalence of carotid calcified plaque. These findings were consistent across age, race/ethnicity, and cigarette smoking, except for an increased prevalence in men with a history of regular cannabis smoking. Similarly, in a subgroup with CAC, no association was found between a history of regular smoking and extent of calcification as measured by volume, density, and Agatston score.

The cardiovascular effects of cannabis are multifaceted, involving both direct and indirect mechanisms mediated by cannabinoid (CB1R and CB2R) and noncannabinoid receptors.^19^ CB1 receptors are predominantly located in the central and peripheral nervous system.^20^ In contrast, CB2 receptors are present on immune cells, and have been identified on macrophages and T lymphocytes within atherosclerotic lesions.^20–22^ Activation of CB1R has been implicated in pro-atherogenic processes such as oxidized LDL formation, endothelial dysfunction, vascular smooth muscle hyperplasia, and induction of inflammatory responses.^22–24^ In contrast, CB2R activation has been associated with antiinflammatory and protective effects against atherosclerosis, including inhibition of macrophage chemotaxis, reduced infiltration and accumulation of monocytes in arterial walls, and downregulation of TH1 immune response leading to inhibition of atherosclerotic progression.^19,21,24–26^ These contradictory actions raise questions about the net impact of regular cannabis use on the development and progression of atherosclerosis.

If smoking cannabis increases the risk of atherosclerotic disease, one would anticipate finding consistent evidence of adverse cardiac outcomes in human research. This is not the case. Some cross-sectional studies have reported associations with increased prevalence of coronary heart disease,^10^ MI,^11,27,28^ and stroke,^12,13,27^ whereas others have reported no association.^29,30^ Longitudinal studies have largely reported no association with CVD risk factors such as inflammation,^31^ abdominal adiposity,^32^ BMI,^33^ blood pressure,^33,34^ lipids,^33^ and glucose,^33,35^ as well as subclinical atherosclerosis,^15^ incident CVD,^36^ and MI,^14,15,31–33,36^ with some exceptions.^37^

These and similar studies may be understood through the lens of a ‘Stoner’s paradox’ in which the common view of cannabis users as sedentary, overweight, unmotivated, and lazy is contradicted by studies revealing higher rates of physical activity,^38^ more favorable BMI profiles,^33,39^ lower concentrations of certain lipid markers,^13,33^ and better glycemic control.^39^ It remains unclear whether these favorable factors counterbalance potential risks.

The null findings reported herein are consistent with our previous research in NHANES which explored associations with MI and blood pressure and hypertension,^29,30^ and with an observational study using longitudinal data from the Coronary Artery Risk Development in Young Adults (CARDIA) study, which reported no association between cumulative cannabis exposure and increased carotid intima–media thickness, including among individuals who never smoked tobacco.^14^ A separate study from CARDIA reported an association between cumulative cannabis exposure and coronary artery calcification, but only among tobacco smokers.^15^

Less than 40% of participants with a history of regular cannabis smoking, reported smoking cannabis in the past month. This finding, consistent with our previous work in NHANES, suggests that most regular use occurs in youth and middle age, potentially indicating a decrease in use over time (‘aging out’). Thus, if recent use, in addition to a history of regular use, underlies the atherosclerotic disease process, abstinence in later life may attenuate risk. This pattern was not seen in our data, however, as participants with a history of both regular use *and* recent use had a lower prevalence of CAC (PR: 1.04, 95% CI: 0.68 to 1.61) compared to those without recent use (PR: 1.19, 95% CI: 0.86 to 1.65), despite having similar ages.

In our NHANES samples of middle-aged adults (35–59 years old; 2009–2018), we observed a history of regular (i.e., monthly) use of approximately 25%, compared to approximately 7% in MESA. As a loose proxy for regular use, 37.6% of participants reported using marijuana 100 times or more at Exam 30 (2015–2016) in the CARDIA cohort (unpublished data). The difference in exposure across these studies is not easily explained.

Given that the NHANES samples and CARDIA cohort were younger, it is possible that changes in attitudes and access to cannabis played a small role (i.e., the changes in attitudes and access may have occurred during formative years, potentially influencing their current usage patterns).

In NHANES, almost 75% of the samples were White individuals, compared to 40% in MESA. It is possible that racial and ethnic minorities are less likely to report illicit drug use in anonymous surveys due to fear of judgment or legal consequences and thus cannabis use is underreported in MESA. Socioeconomic status is unlikely to explain the difference, given that the NHANES samples were more educated with higher income. Further, the days used in the past month among participants with a history of regular use were approximately 15 in NHANES and almost 17 in MESA. Thus, though the frequency may be similar, the prevalence of exposure is not.

The strengths of our study include the use of a racially and ethnically diverse sample, a relatively large sample size, a specified method of cannabis use (smoking), and the use of a standardized objective outcome measure. The focus on history of regular smoking, including the duration and recency of smoking, is also a strength, given that repeated exposure over time, rather than recent use, is likely to be the primary contributor to chronic CVD. Furthermore, the analysis of calcium volume and density, alongside the Agatston score, was conducted as a strategy to enhance understanding of the potential mechanisms, should greater prevalence have been identified.

Limitations include the cross-sectional design, average age of participants (> 74 years), high attrition of MESA participants from enrollment to assessment of cannabis use and CAC at Exam 6, self-reported exposure of a federally illegal substance, and the relatively small number of MESA participants who reported a history of regular cannabis smoking (*n* = 159, 7.4%) and a history of recent cannabis smoking (*n* = 60, 2.8%) relative to other US estimates,^30^ which may limit the statistical power and generalizability of our findings. Further, though CAC is a marker of atherosclerosis, noncalcified plaques are also associated with cerebral events. By focusing on advanced atherosclerosis and not earlier stage disease, the study may have failed to detect existing disease that has not progressed to calcification. Our study aimed to extend the analysis to stroke outcomes, but limited data availability restricted our analysis. A history of stroke was recorded for only 66 participants in the dataset. Of which, four (2.5%) occurred in participants with a history of regular cannabis smoking and 62 (3.1%) occurred in those without a history.

Notably, many cross-sectional studies reporting positive associations with CVD risks have focused on recency of use (past month) as the measure of cannabis exposure. Though ‘temporality’ is a potential limitation of the cross-sectional design, it is impossible to infer that the exposure preceded the event when the exposure is limited to the past 30 days. Hence, such findings should be interpreted with caution. Additionally, the inability to visualize the entire carotid artery on lung CT scans may have limited our ability to fully assess the extent of carotid artery calcification.

Despite these null findings, and given the conflicting results of comparable studies, there is a critical need for prospective cohort studies examining associations between patterns of cannabis use over time and adverse cardiovascular outcomes, including calcified arterial plaque. These studies should include an analysis of the duration and frequency of cannabis use, as well as an examination of the method of use, specifically comparing combusted (i.e., smoked) versus noncombusted forms and inhaled versus noninhaled routes of administration.

## Conclusion

In a racially and ethnically diverse cohort of older adults with a moderately high prevalence of carotid artery calcification, there were no statistically significant associations between a history of regular cannabis smoking (primary analysis), duration, or recency of cannabis smoking, and the prevalence of carotid calcified plaque. These findings were consistent across age, race/ethnicity, and cigarette smoking, except for an increased prevalence in men with a history of regular cannabis smoking. Similarly, in a subgroup with carotid artery calcification, no association was found between a history of regular smoking and extent of calcification as measured by volume, density, and Agatston score.

## Supplemental Material

sj-pdf-1-vmj-10.1177_1358863X241287690 – Supplemental material for Regular cannabis smoking and carotid artery calcification in the Multi-Ethnic Study of Atherosclerosis (MESA) Supplemental material, sj-pdf-1-vmj-10.1177_1358863X241287690 for Regular cannabis smoking and carotid artery calcification in the Multi-Ethnic Study of Atherosclerosis (MESA) by Jamie Corroon, Ryan Bradley, Igor Grant, Michael R Daniels, Julie Denenberg, Michael P Bancks and Matthew A Allison in Vascular Medicine
