# Supplementary material for: Regular cannabis smoking and carotid artery calcification in the Multi-Ethnic Study of Atherosclerosis (MESA)
Source: Vasc Med. 2024 Oct 23;29(6):621–31. doi: 10.1177/1358863X241287690 (PMC11590377; doi:10.1177/1358863X241287690)
Supplement: sj-pdf-1-vmj-10.1177_1358863X241287690 – Supplemental material for Regular cannabis smoking and carotid artery calcification in the Multi-Ethnic Study of Atherosclerosis (MESA) [file sj-pdf-1-vmj-10.1177_1358863X241287690.pdf]

SUPPLEMENTAL MATERIAL: Regular cannabis smoking and its relationship to carotid artery calcification in the Multi-Ethnic Study of Atherosclerosis (MESA), Corroon et al.

Jamie Corroon, ND, MPH[1]
Igor Grant MD, FRCP(C)[2]
Ryan Bradley, ND, MPH[3]
Matthew A Allison, MD, MPH, FAHA[1]

[1] Department of Family Medicine, University of California San Diego, La Jolla, CA.
[2] Center for Medicinal Cannabis Research, Department of Psychiatry, University of California San Diego, La Jolla, CA.
[3] Herbert Wertheim School of Public Health and Human Longevity Science, University of California San Diego, La Jolla, CA.

Corresponding author: Jamie Corroon, ND, MPH (jcorroon@health.ucsd.edu)

Table of Contents

SUPPLEMENTARY METHODS 2
Composite exposure 2
Covariates 2
Stratified Analyses 2
Carotid Artery Calcium (CAR) Scoring Methodology 2
SUPPLEMENTARY RESULTS 4
Figure 1 Supplemental – Flow diagram of study sample selection 4
Table 1 Supplemental – Adjusted prevalence ratios and absolute prevalence differences for calcified plaque in either carotid artery by cannabis smoking characteristics 5
Table 2 Supplemental – Adjusted beta coefficients and 95% confidence intervals for extent of calcified plaque in carotid arteries by cannabis smoking characteristics 6

SUPPLEMENTARY METHODS

Composite exposure

A composite measure was created (i.e., joint/pipe years) to assess a multifaceted exposure including duration, frequency, and quantity of cannabis smoking. Joint/pipe years was calculated using the low end, midpoint, and high end for each categorical response to “During the time that you smoked marijuana or hashish regularly, how often would you usually smoke it?”. For the midpoint, joint/pipe years was calculated as follows: (midpoint value of each categorical response to “During the time that you smoked marijuana or hashish regularly, how often would you usually smoke it?” x 12 months/year x “On the days that you smoked marijuana or hashish, how many joints or pipes would you usually smoke?” x years of regular smoking) / days per year (For example: (6 times per month x 12 months/year x 3 joints/pipes per day x 10 years of regular smoking) / 365.25 = 5.9 joint/pipe years. Midpoint values were determined as follows: “Missing”=0, “once per month” =1, “2-3 time per month” =2.5, “4-8 times per month (about 1-2 times per week)”=6, “9-24 times per month (about 3-5 times per week)”=16.5, “25-30 times per month (about one or more times per day)” = 27.5 joint/pipe years.

Covariates

Sex, race/ethnicity, household income and educational attainment were assessed using standard questionnaires at the baseline visit.[1] Age, physical activity, cigarette smoking, current alcohol use, diabetes status, and medication use were assessed using standard questionnaires at Exam 6. Physical activity was reported as the total of moderate and vigorous activity, measured in MET-min/week from Monday through Sunday. Values exceeding the 99th percentile (n=31) were deemed unrealistic and were set equal to the 99th percentile (27,870 MET-min/week).

Stratified Analyses

Stratified analyses were used to assess differences between a history of regular cannabis smoking and the prevalence of CAR in age-defined subgroups (i.e., 59-69, 70-80, > 80 years), race/ethnicity-defined subgroups (i.e., Non-Hispanic White, Non-Hispanic Black, Hispanic), subgroups by biologic sex (i.e., male, female), and subgroups based on cigarette smoking status (i.e., current, former, never). For race/ethnicity, Chinese participants were excluded due to insufficient data. Multiplicative first-order interactions were constructed for each covariate in the final model. Interaction terms were considered significant in the final multivariable-adjusted model if $p < 0.10$.

Carotid Artery Calcium (CAR) Scoring Methodology

The Agatston scoring method, originally developed for coronary artery calcium quantification,[2] has been extended to calcium assessment and measurement in multiple arterial beds, including the carotid arteries.[3-7] While a high Agatston score in non-coronary arteries is a marker of increased cardiovascular risk, its clinical implications are still being explored, and further studies are needed to fully understand its significance.

Methodology:

1. **Image Acquisition**: CT scans from Exam 6 were transferred from the MESA Reading Center to UCSD for CAR interpretation and scoring.

2. **Image Analysis**: For quantification of the calcium burden, all images were transferred to an independent PC-based workstation with the Philips Extended Brilliance Workspace (EBW), a multimodality platform for advanced visualization and analysis.

3. **Calcification Identification**: Plaques were defined as three contiguous pixels with a density > 130 Hounsfield Units (HU).

4. **Scoring**: Regions of interest (ROIs), plaques were identified by analyzing the bilateral external carotid arteries. The Philips's Software uses The Area/Step method, based on Agatston score, which includes the Area of the calcified lesion and the average CT value within the lesion. The volume and the weighing factor are continuous.
   a. The lesion **volume** is obtained by multiplying the area (in mm2) that is above the threshold – the minimum CT in the 1 Step/Weight factor - by the distance between reconstructed slices (in mm). The continuous weight factor (WC), for calcified lesions above the threshold, is calculated by the formula: Wc= (CT-50) / 100
   b. The Volumetric score is calculated as follows: Score = (Wc) x (the lesion volume)
   c. The Score of all ROIs attained for all images is summed. This sum is the Total **Score** for the segment of interest.
   d. The Philips score was changed into a density score in Hounsfield units (HU) using the conversion factor - ((**Score/Volume)***100)+50 for the segment of interest. A Hounsfield units score enabled the data to be compared to relevant studies.

5. **Reliability**: Scoring was primarily performed by a qualified operator (Mike Daniels, Staff Research Associate, UCSD) with extensive training and experience. The intra-rater reliability correlation coefficients for the Agatston, volume, and mean and max HU scores were all very high, ranging from 0.98 to 0.93. Only the region of interest variables on the left and right sides were less than 0.93 (0.85 and 0.64, respectively).

6. **Data Management**: CT images and scores were securely stored and managed on a HIPAA-compliant, password-protected server at UCSD.

This methodology provides a standardized approach for quantifying carotid artery calcification, allowing for consistent assessment across study participants.

SUPPLEMENTARY RESULTS

Figure 1 Supplemental – Flow diagram of study sample selection

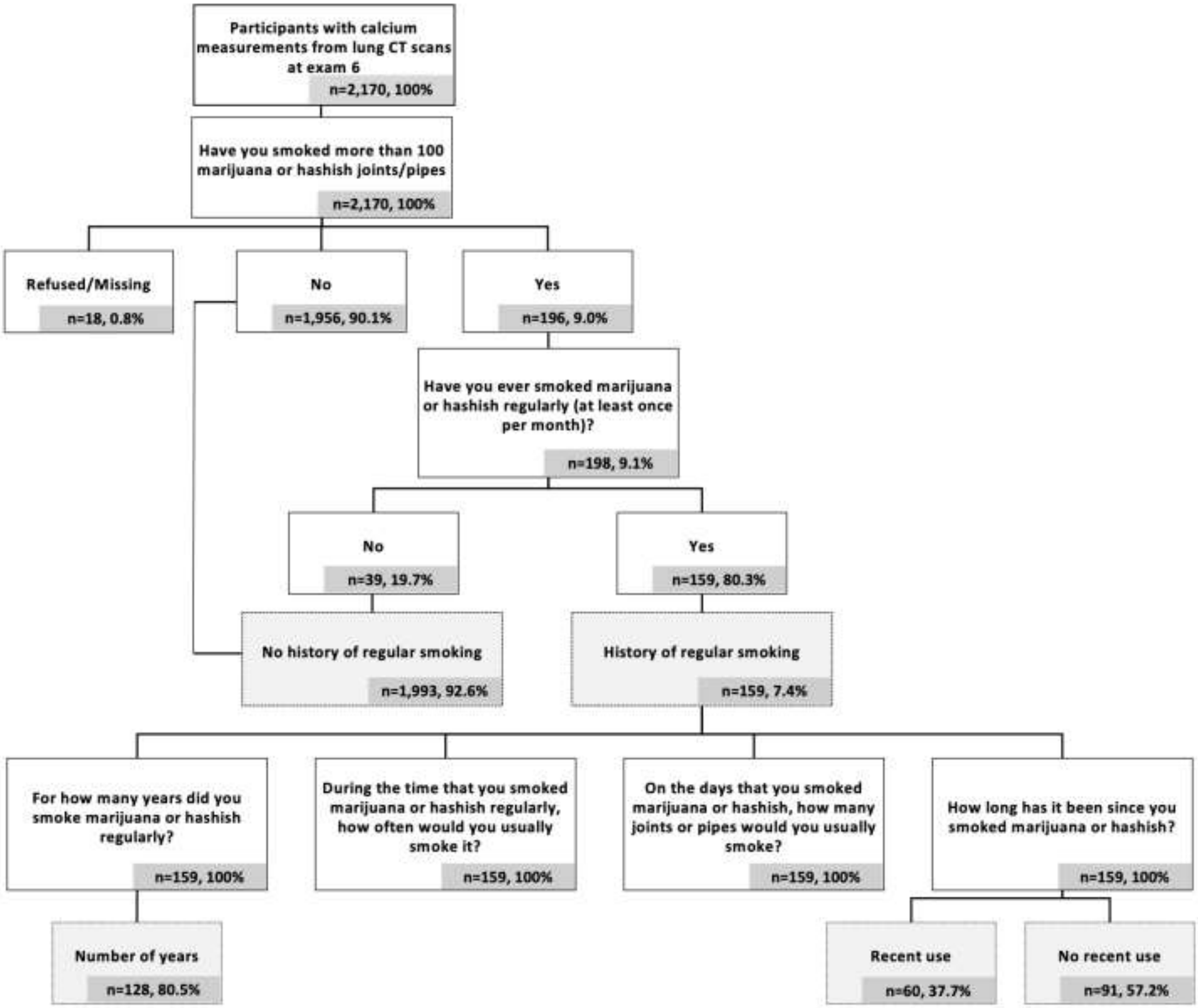

Table 1 Supplemental – Adjusted prevalence ratios and absolute prevalence differences for calcified plaque in either carotid artery by cannabis smoking characteristics

Cannabis characteristic		Unadjusted		Model 1	
	Count/Count (Prevalence)	Prevalence Ratio	Absolute Prevalence Difference	Prevalence Ratio	Absolute Prevalence Difference
**History of regular smoking**		(n=2,152)		(n=2,071)	
No	728/1990 (36.6%)	**1.00 Ref.**	**0.00 Ref.**	**1.00 Ref.**	**0.00 Ref.**
Yes	48/159 (30.2%)	0.83 (0.65 to 1.05)	-6.4 (-13.8 to 1.1)	1.23 (0.97 to 1.56)	7.5 (0.1 to 15.0)
**Duration of regular smoking, years**		(n=2,120)		(n=2,039)	
0	728/1990 (36.6%)	**1.00 Ref.**	**0.00 Ref.**	**1.00 Ref.**	**0.00 Ref.**
1 to 5	14/47 (29.8%)	0.81 (0.52 to 1.27)	-6.8 (-20.0 to 6.5)	1.26 (0.82 to 1.95)	8.7 (-4.4 to 21.7)
> 5 to 10	7/26 (26.9%)	0.74 (0.39 to 1.39)	-9.7 (-26.8 to 7.5)	1.28 (0.80 to 2.06)	5.9 (-9.9 to 21.7)
> 10	17/54 (31.5%)	0.86 (0.58 to 1.28)	-5.1 (-17.7 to 7.5)	1.28 (0.91 to 1.80)	7.0 (-4.8 to 18.7)
**Recency of smoking**		(n=2,144)		(n=2,063)	
No history of regular smoking	728/1990 (36.6%)	**1.00 Ref.**	**0.00 Ref.**	**1.00 Ref.**	**0.00 Ref.**
No past month smoking	30/91 (33.0%)	0.90 (0.67 to 1.21)	-3.6 (-13.5 to 6.3)	1.38 (1.05 to 1.80)	9.9 (0.4 to 19.3)
Past month smoking	15/60 (25.0%)	0.68 (0.44 to 1.06)	-11.6 (-22.7 to -0.4)	1.06 (0.70 to 1.60)	3.6 (-7.7 to 14.8)

Cannabis characteristic	Model 2		Fully Adjusted	
	Prevalence Ratio	Absolute Prevalence Difference	Prevalence Ratio	Absolute Prevalence Difference
**History of regular smoking**	(n=2,045)		(n=1,997)	
No	**1.00 Ref.**	**0.00 Ref.**	**1.00 Ref.**	**0.00 Ref.**
Yes	1.13 (0.88 to 1.46)	5.7 (-2.0 to 13.4)	1.14 (0.88 to 1.49)	4.7 (-2.7 to 12.0)
**Duration of regular smoking, years**	(n=2,014)		(n=1,967)	
0	**1.00 Ref.**	**0.00 Ref.**	**1.00 Ref.**	**0.00 Ref.**
1 to 5	1.25 (0.81 to 1.93)	8.5 (-4.3 to 21.3)	1.35 (0.91 to 2.01)	7.9 (-4.0 to 19.8)
> 5 to 10	1.30 (0.73 to 2.31)	4.5 (-11.8 to 20.8)	1.42 (0.90 to 2.23)	4.6 (-10.0 to 19.3)
> 10	1.05 (0.72 to 1.54)	2.9 (-9.5 to 15.3)	1.00 (0.64 to 1.56)	2.1 (-10.4 to 14.7)
**Recency of smoking**	(n=2,037)		(n=1,989)	
No history of regular smoking	**1.00 Ref.**	**0.00 Ref.**	**1.00 Ref.**	**0.00 Ref.**
No past month smoking	1.30 (0.97 to 1.74)	8.2 (-1.5 to 17.9)	1.19 (0.86 to 1.65)	6.3 (-2.9 to 15.4)
Past month smoking	0.93 (0.62 to 1.39)	1.5 (-10.2 to 13.1)	1.04 (0.68 to 1.61)	1.2 (-10.3 to 12.7)

Model 1: site, age, gender, race/ethnicity, HH income.
Model 2: Model 1 plus alcohol and cigarette use, physical activity
Model 3 : Model 2 plus BMI, total cholesterol to HDL cholesterol ratio, FBG, antihypertensive, antilipidemic, and antidiabetic medications.
Abbreviations: CI, confidence interval; HH, household; BMI, body mass index; HDL, high density lipoprotein; FBG, fasting blood glucose
p-values for multiple comparisons have been adjusted using Tukey's method.
Bold = p < 0.05

SUPPLEMENTAL MATERIAL: Regular cannabis smoking and its relationship to carotid artery calcification in the Multi-Ethnic Study of Atherosclerosis (MESA), Corroon et al.

Table 2 Supplemental – Adjusted beta coefficients and 95% confidence intervals for extent of calcified plaque in carotid arteries by cannabis smoking characteristics

Cannabis characteristic	Unadjusted			Model 1		
	Volume (mm3)	Density (HU)	Agatston score	Volume (mm3)	Density (HU)	Agatston score
**History of regular smoking (y/n)**						
Left common carotid	22.8 (-33.8 to 127.8) (n=447)	3.0 (-13.1 to 22.1) (n=447)	26.1 (-41.9 to 173.7) (n=447)	62.6 (-14.0 to 207.5) (n=428)	13.9 (-4.5 to 35.8) (n=428)	86.2 (-16.3 to 313.8) (n=428)
Right common carotid	-13.5 (-50.7 to 51.7) (n=625)	-0.7 (-13.0 to 13.3) (n=625)	-19.7 (-59.4 to 58.6) (n=625)	9.5 (-38.6 to 95.4) (n=595)	6.2 (-7.7 to 22.1) (n=595)	7.3 (-46.9 to 116.9) (n=595)
Either common carotid	-11.0 (-46.9 to 49.0) (n=777)	0.0 (-10.7 to 12.0) (n=777)	-14.4 (-54.0 to 59.5) (n=777)	24.4 (-26.4 to 110.1) (n=741)	8.1 (-3.8 to 21.5) (n=741)	30.3 (-30.8 to 145.2) (n=741)
**Duration of regular smoking, years**						
Either common carotid	-0.2 (-1.7 to 1.2) (n=731)	0.2 (-0.1 to 0.5) (n=731)	1.9 (-1.0 to 5.0) (n=731)	1.4 (-1.1 to 3.9) (n=731)	0.5 (-0.1 to 1.0) (n=731)	1.7 (-1.3 to 4.7) (n=731)
**Joint/pipe years, mid**						
Either common carotid	1.1 (-0.4 to 2.7) (n=760)	1.5 (0.0 to 3.1) (n=760)	1.3 (-0.6 to 3.2) (n=760)	1.5 (0.0 to 3.1) (n=760)	0.2 (-0.1 to 0.6) (n=760)	1.8 (0.0 to 3.7) (n=760)

Cannabis characteristic	Model 2			Model 3			Fully Adjusted		
	Volume (mm3)	Density (HU)	Agatston score	Volume (mm3)	Density (HU)	Agatston score	Volume (mm3)	Density (HU)	Agatston score
**History of regular smoking (y/n)**									
Left common carotid	7.8 (-26.6 to 58.4) (n=428)	3.2 (-7.0 to 14.5) (n=428)	84.7 (-17.6 to 314.0) (n=428)	13.6 (-23.3 to 68.2) (n=421)	1.7 (-8.6 to 13.2) (n=421)	88.0 (-17.5 to 328.4) (n=421)	13.7 (-23.1 to 68.2) (n=415)	1.4 (-8.9 to 12.8) (n=415)	81.4 (-20.4 to 313.5) (n=415)
Right common carotid	-1.2 (-29.9 to 39.3) (n=595)	2.3 (-5.6 to 10.9) (n=595)	20.8 (-40.2 to 143.9) (n=595)	-1.9 (-30.5 to 38.4) (n=588)	2.0 (-6.0 to 10.7) (n=588)	12.8 (-44.5 to 129.3) (n=588)	10.9 (-22.2 to 58.1) (n=574)	-1.5 (-9.4 to 7.1) (n=574)	12.2 (-47.0 to 137.4) (n=574)
Either common carotid	-1.9 (-28.4 to 34.5) (n=741)	3.4 (-3.6 to 10.9) (n=741)	41.1 (-25.2 to 166.3) (n=741)	-2.3 (-28.7 to 33.9) (n=732)	3.0 (-4.0 to 10.6) (n=732)	32.1 (-30.3 to 150.3) (n=732)	7.7 (-21.8 to 48.5) (n=715)	0.4 (-6.6 to 7.9) (n=715)	32.1 (-31.8 to 155.8) (n=715)
**Duration of regular smoking, years**									
Either common carotid	-0.2 (-1.7 to 1.3) (n=731)	0.2 (-0.1 to 0.6) (n=731)	1.7 (-1.3 to 4.7) (n=731)	-0.5 (-2.0 to 0.9) (n=722)	0.2 (-0.1 to 0.6) (n=722)	0.9 (-2.0 to 3.9) (n=722)	0.0 (-1.4 to 1.5) (n=706)	0.1 (-0.2 to 0.4) (n=706)	1.5 (-1.6 to 4.6) (n=706)
**Joint/pipe years, mid**									
Either common carotid	0.6 (-0.3 to 1.5) (n=724)	0.0 (-0.2 to 0.2) (n=724)	1.9 (0.1 to 3.8) (n=724)	0.6 (-0.3 to 1.4) (n=715)	0.0 (-0.2 to 0.2) (n=715)	1.7 (-0.1 to 3.5) (n=715)	0.7 (-0.2 to 1.6) (n=700)	-0.1 (-0.3 to 0.1) (n=700)	1.7 (-0.1 to 3.5) (n=700)

Percent change in volume, density, and Agatston score were calculated after natural log transformation and then back-transformed.
Model 1: age, gender, race/ethnicity, HH income.
Model 2: Model 1 plus either Volume (mm3) or Density (HU)
Model 3: Model 2 plus alcohol and cigarette use, physical activity
Fully adjusted model: Model 3 plus BMI, SBP, DBP, total cholesterol to HDL cholesterol ratio, FBG, antihypertensive, antilipidemic, antidiabetic medications and total medications.
Abbreviations: CI, confidence interval; HH, household; BMI, body mass index; HDL, high density lipoprotein; FBG, fasting blood glucose; HU, Hounsfield Units
Note: Cannabis characteristics were natural log transformed. The resulting coefficients were exponentiated for presentation. The line of no difference for confidence intervals is 1.0.
Bold = p < 0.05

References

1. DE B, DA B, GL B, et al. Multi-Ethnic Study of Atherosclerosis: objectives and design. *American journal of epidemiology*. 11/01/2002 2002;156(9):871-881. doi:10.1093/aje/kwf113
2. Agatston AS, Janowitz Wr Fau - Hildner FJ, Hildner Fj Fau - Zusmer NR, Zusmer Nr Fau - Viamonte M, Jr., Viamonte M Jr Fau - Detrano R, Detrano R. Quantification of coronary artery calcium using ultrafast computed tomography. (0735-1097 (Print))
3. Thomas IC, McClelland RL, Michos ED, et al. Density of calcium in the ascending thoracic aorta and risk of incident cardiovascular disease events. (1879-1484 (Electronic))
4. MH C, JO D, RL M, et al. Abdominal aortic calcium, coronary artery calcium, and cardiovascular morbidity and mortality in the Multi-Ethnic Study of Atherosclerosis. *Arteriosclerosis, thrombosis, and vascular biology*. 2014 Jul 2014;34(7)doi:10.1161/ATVBAHA.114.303268
5. MA A, S H, CL W, et al. Calcified atherosclerosis in different vascular beds and the risk of mortality. *Arteriosclerosis, thrombosis, and vascular biology*. 2012 Jan 2012;32(1):140–146. doi:10.1161/ATVBAHA.111.235234
6. Hyder JA, Allison Ma Fau - Wong N, Wong N Fau - Papa A, et al. Association of coronary artery and aortic calcium with lumbar bone density: the MESA Abdominal Aortic Calcium Study. (1476-6256 (Electronic))
7. H B, P B, Z M, et al. Association of coronary artery calcification and thoracic aortic calcification with incident peripheral arterial disease in the Multi-Ethnic Study of Atherosclerosis (MESA). *European heart journal open*. 12/03/2021 2021;1(3)doi:10.1093/ehjopen/oeab042
